# Measuring Place Connectivity Using Big Social Media Data

**Published:** 2021-02-08

**Authors:** Zhenlong Li, Xiao Huang, Xinyue Ye, Yuqin Jiang, Martin Yago, Huan Ning, Michael E. Hodgson, Xiaoming Li

**Affiliations:** 1 Geoinformation and Big Data Research Laboratory, Department of Geography, University of South Carolina, SC, USA; 2 Department of Geosciences, University of Arkansas, AR, USA; 3 Department of Landscape Architecture & Urban Planning, Texas A&M University, TX, USA; 4 School of Public Administration, University of Central Florida, FL, USA; 5 Department of Health Promotion, Education, and Behavior, University of South Carolina, SC, USA

**Keywords:** place connectivity, spatial interaction, big data, Twitter, SafeGraph, Facebook SCI

## Abstract

Shaped by human movement, place connectivity is quantified by the strength of spatial interactions among locations. For decades, spatial scientists have researched place connectivity, applications, and metrics. The growing popularity of social media provides a new data stream where spatial social interaction measures are largely devoid of privacy issues, easily assessable, and harmonized. In this study, we introduced a place connectivity index (PCI) based on spatial interactions among places revealed by geotagged tweets as a multiscale, spatiotemporal-continuous, and easy-to-implement measurement. The proposed PCI, established and demonstrated at the US county level, exhibits a strong positive association with SafeGraph population movement records (10% penetration in the US population) and Facebook’s social connectedness index (SCI), a popular connectivity index based on social networks. We found that PCI has a strong state boundary effect and that it generally follows the distance decay effect, although this force is weaker in more urbanized counties with a denser population. Our investigation further suggests that PCI has great potential in addressing real-world problems that require place connectivity knowledge, exemplified with two applications: 1) modeling the spatial spread of a contagious disease (e.g., COVID-19), and 2) modeling hurricane evacuation destination choices. The methodological and contextual knowledge of PCI, together with the launched visualization platform and data sharing capability, is expected to support research fields requiring knowledge in human spatial interactions.

## Introduction

Since the proposal of “social physics” in 1948 by John Stewart, an astrophysicist who first attempted to reveal spatial interaction based on the concept of the Newtonian gravitational framework (Stewart, 1948), research on modeling, documenting, and understanding human spatial interaction has been a research hotspot in geography and related fields. From a geographic perspective, human movements express the spatial interactions among places, featured by human characteristics (population, land use, culture, etc.) and physical characteristics (climate, geology, landscape, etc.) (Massey, 1994). Relationships among places are shaped by constant human movement, and the intensity of such movement further quantifies the connectivity strength among places. Thus, understanding connectivity between two places provides fundamental knowledge regarding their interactive gravity, benefiting various applications such as infectious disease modeling, transportation planning, tourism management, evacuation modeling, and other fields requiring knowledge in human spatial interactions.

However, measuring such interactions is a challenging task. For decades, geographers have been trying to document spatial interactions and place connectivity at various spatiotemporal scales. Early efforts (widely adopted until now) to examine spatial interactions involved survey utilization. Researchers used questionnaires to understand spatial interactions, aiming to gauge both long-term spatial movement, such as migration patterns (Barcus and Brunn, 2010; Boyle, 2014; Salt, 1987;), and short-term spatial displacement, such as evacuation and traveling (Buliung and Kanaroglou, 2006; Calantone et al., 1989; Durage et al., 2014; Pham et al., 2020; Santos et al., 2011; Siebeneck and Cova, 2012). The well-documented spatial interactions from these surveys contribute to our understanding of how people move across space and how places are connected; however, such an approach has been criticized because of small sample sizes (Martín et al., 2020a), limited temporal resolution (Pereira et al., 2013), and resource demands (Martín et al., 2020b).

The limitations of survey-based approaches largely preclude temporal-continuous observations in spatial interactions, therefore inducing discrete place connectivity measurements. However, place connectivity should not be considered as a fixed spatiotemporal property of places. Instead, connectivity is ever-changing and evolving rapidly in modern society (Batten, 2001; Gao, 2015; Macdonald and Grieco, 2007). As argued by many, technological advances in the past decades have greatly facilitated connectivity by the weakening geographic limits (Tranos and Nijkamp, 2013). To capture the temporal nature of spatial interactions, researchers have emphasized the importance of transportation data that detail people’s moving patterns. Place connectivity has been measured using various transportation means that include airline flows (Derudder and Witlox, 2008; Xu and Harriss, 2008), highway traffic (Zhang et al., 2020), railway flows (Yang et al., 2019), and intercity bus networks (Ghafoor et al., 2014). The rich traffic information and the derived spatial networks greatly facilitate our understanding of how places are connected via these transportation modes. However, transportation-based approaches pose new challenges. First, such data are generally difficult, as they are often confidential or collected by private companies. Second, the data themselves are mode-specific, lacking the holistic views of the overall human spatial interactions and place connectivity, which are often needed in fields such as infectious disease modeling.

The emerging concepts of “Web 2.0” (O’Reilly, 2007) and “Citizen as Sensors” (Goodchild, 2007), largely benefiting from the advent of geopositioning technologies, offer a new avenue to actively and passively gather and collect the digital traces left by electronic device holders (Li et al., 2019; Martin et al., 2020b). For example, passive trace collection involves data obtained from mobile phone data (Amini et al., 2014; Gonzalez et al., 2008), smart cards (Agard et al., 2006; Ma et al., 2013), or wireless networks (Perttunen et al., 2014). The spatial interactions documented from these passively collected traces tend to have high representativeness, given their high data penetration ratios. However, privacy and confidentiality concerns have been raised for such approaches, as individuals do not intend to actively share their locational information and are unaware of the usage of the generated positions (de Montjoye et al., 2018; Kontokosta and Johnson, 2017).

An approach less encumbered with privacy issues is based on spatial information from social media, a digital platform aiming to facilitate information sharing that has been popularized in recent years. Owing to their active sharing characteristics, social media data (e.g., Twitter, Facebook, and Instagram) are less abundant compared to passively collected GPS positions from mobile devices but are less intrusive (Huang et al., 2020a; Jiang et al., 2019a), more accessible (Hu et al., 2020), and more harmonized (Huang et al., 2020b). The huge volume of user-generated content covering extensive areas facilitates the timely need for summarizing human spatial interactions. Twitter, for example, has quickly become the largest social media data source for geospatial research and has been widely used in human mobility studies (Fiorio et al., 2017; Jurdak et al., 2015; Li et al., 2020a; Soliman et al., 2017), given its free application programming interface (API) that allows unrestricted access to about 1% of the total tweets (Hawelka et al., 2014). We believe that the enormous sensing network constituted by millions of social media users worldwide provides unprecedented data to understand place connectivity at various spatiotemporal scales.

As an essential component in human interaction, social connections that involve online friendships, account following, and information reposting can also contribute to place connectivity measurement. A recent effort from Facebook explores connectivity measurement among places (called Social Connectedness Index, SCI) utilizing the social networks constructed from massive friendship links on Facebook (Bailey et al., 2018). However, whether or how the place connectivity measured by social connections differs from the one measured by physical connections is unknown. Gaps still exist in 1) current literature on how a harmonized, multiscale, spatiotemporal-continuous place connectivity measurement based on population movement can be constructed, 2) the utility of place connectivity metrics in addressing real-world problems, and 3) a tool to visualize connectivity and provide downloadable connectivity matrices to support research needs.

Taking advantage of big social media data and the advancement of high-performance computing, we proposed a place connectivity index (PCI) based on people’s movement among places captured from big social media data. Specifically, in this study, we computed PCI from geotagged tweets at the US county level. We compared population movement derived from Twitter data with the SafeGraph (2020) movement data to evaluate how well geotagged tweets captured population movement at the county level. We compared PCI with Facebook’s SCI, a popular connectivity index based on social networks, to reveal the association between spatial interactions and social interactions. We also investigated the spatial properties of PCI and found that PCI has a strong state boundary effect and that it generally follows the distance decay effect, although this effect is weaker in more urbanized counties with denser populations.

The utility of PCI is demonstrated in two applications: 1) modeling the spatial spread of COVID-19 during the early stage of the pandemic, and 2) modeling hurricane evacuation destination choice. The results demonstrate the great potential of PCI in addressing real-world problems requiring place connectivity knowledge. Finally, we constructed massive PCI matrices and launched an interactive portal for users to visualize the strength of connectivity among geographic regions at various scales. The derived PCI matrices among user-defined geographical regions are open-sourced and downloadable to support research needs. Serving as a harmonized and understandable connectivity metric, the proposed PCI is expected to benefit varied domains demanding place connectivity knowledge, such as disease transmission modeling, transportation planning, evacuation simulation, and tourist prediction.

## Conceptualization of Place Connectivity Index

**Fig. 1.**
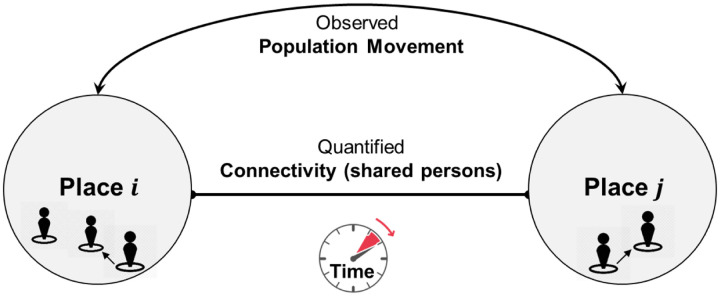
Conceptualization of Place Connectivity Index with population movement in a space-time framework

We define a Place Connectivity Index (PCI) between two places as the normalized number of shared persons (e.g., unique Twitter users) between the two places during a specified time period (e.g., one year; Fig. 1). For example, if a user is observed at both places during the time period, the user is considered a shared user between the two places. PCI can be computed at various geographic scales. For example, a place can be a county, state, or country. PCI does not aim to capture the real-time population movement between places (though it is derived from such movement); rather, it provides a relatively stable measurement of how strong two places are connected by spatial interactions. The strength of the connection between two places can be determined by many factors, such as geographic distance (the first law of geography; Miller, 2004), transportation, administrative (regional) limits (e.g., states), physical barriers (e.g., rivers/mountains), social networks, demographic and socioeconomic similarities or differences. In this sense, PCI should be calculated in a relatively long time period (e.g., a year) to gather sufficient information to summarize the general patterns.

Specifically, the PCI between place *i* and place *j* (denoted as *PCI*_*ij*_) can be computed by Eq. 1.

Eq. 1 $$PCI_{ij}\;=\;\sqrt{\frac{S_{ij}{}^{2}}{S_{i}\;S_{j}}}\;i,\;j\;\in \;\left[1,\;n\right]$$

where *S*_*i*_ is the number of observed persons (social media users) in place *i* within time period *T*; *S*_*j*_ is the number of observed persons in place *j* within time period *T*; *S*_*ij*_ is the number of shared persons between places *i* and *j* within time period *T;* and *n* is the number of places in the study area.

The denominator in Eq. 1 is used to normalize the metric based on the relative populations in the two places. Places with a higher population tend to have more social media users, and thus tend to have more shared users among them. PCI ranges from 0 to 1. When no shared user is observed between two places, PCI equals 0. If all users in place *i* visit place *j* (vice versa) and the two places have the same number of users (or when *i = j*), PCI equals 1. With normalization, PCI provides a relative measurement of how strong places are connected through human spatial interactions when assuming all places have the same population (social media users). This allows us to compare PCI among different places to reveal potential spatial, population, and socioeconomic structures.

**Fig. 2.**
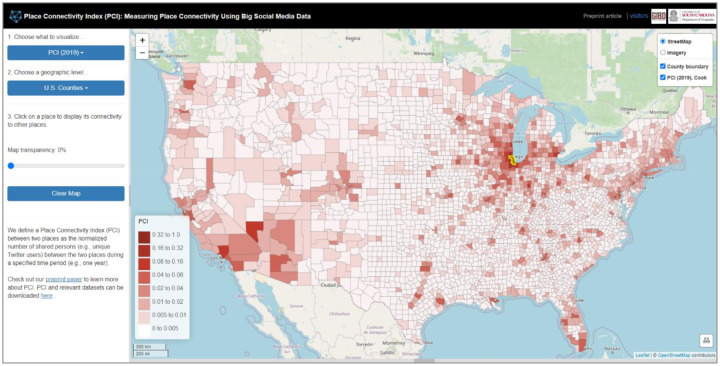
Interactive web portal for PCI visualization. The map shows the PCI for Cook County (Chicago), Illinois, to all other counties. The portal is publicly accessible at http://gis.cas.sc.edu/GeoAnalytics/pci.html

US county-level PCI in 2019 was computed for each county pair (4,832,940 possible county pairs with 3,109 contiguous US counties) with Eq. 1 using 391,503,203 geotagged tweets from 4,892,458 distinct Twitter users in a high-performance computing environment (see Appendix A for computation steps). As not all county pairs exhibited a shared user, the total observed county pairs were 3,405,113. An interactive web portal was developed to visualize a single county’s PCI measured connectivity to other counties (Fig. 2). The number of shared users among county pairs (without normalization) was also included in the portal for comparison with PCI.

## Evaluation

### Comparing with SafeGraph Population Movement

One of the key concerns of using social media data (e.g., Twitter) for human mobility studies is its low population penetration rate. For example, only 24% of US adults use Twitter (Pew Research Center, 2019), and the public Twitter API only returns about 1% of the whole Twitter streams. Also, Twitter data show bias in its representativeness of population groups. This issue has been examined in a few studies (Jiang et al., 2019; Li et al., 2013; Malik et al., 2015). In light of these issues, it is important to evaluate how well geotagged tweets capture population movements (at the county level in this study) since PCI is computed from such movement. For this purpose, we compared the US county-level population movement derived from Twitter to the movement derived from SafeGraph (https://www.safegraph.com), a commercial data company that aggregates anonymized location data from various sources. According to SafeGraph (2019), the data are aggregated from about 10% of mobile devices (e.g., cellphones) in the US, and the sampling correlates highly with the actual US Census populations, with a Pearson correlation coefficient *r* of 0.97 at the county level. Specifically, the data we used in this study are the publicly available SafeGraph’s Social Distancing Metrics (SDM) (SafeGraph, 2020), a census block group level daily mobility data product going back to January 1, 2019 covering the entire US. Since these data only provide aggregated mobility information, deriving the shared users among counties is not possible. Alternatively, we computed the total number of person-day movements between all contiguous US county pairs in 2019 using the SDM (see Appendix B). To make it comparable, we also computed the total number of person-day movements between all US county pairs in 2019 using Twitter data (see Appendix C). We then compared, using Pearson’s *r*, the two person-day movement datasets by county.

**Fig. 3.**
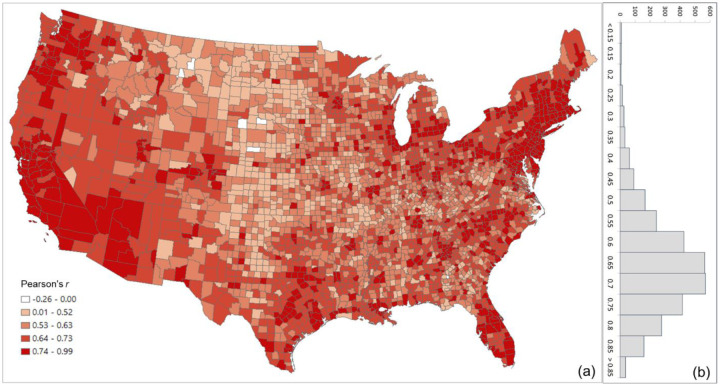
Distribution of the Pearson’s r between the log Twitter person-day movements and log SafeGraph person-day movements for all counties (a) Spatial distribution; (b) histogram

The overall Pearson’s *r* for all county pairs (*n* = 1,516,210) between log Twitter person-day movements and log SafeGraph person-day movements is 0.71. The rationale for using log transformation (with base 10) is to address the highly skewed distribution of movements among counties. To reveal the spatial variations of the relationship for different areas, we further evaluated the association between the two movement datasets for the county pairs from each county to other counties. The spatial distribution of *r* illustrates lower values generally clustering in less populated areas, such as the Great Plains portion of the US (Fig. 3a). This is as expected, as Twitter data generally suffer in less populated areas due to insufficient tweets collected using the public free API. The histogram (Fig. 3b) indicates the most repeated *r* ranges between 0.65 and 0.75.

**Fig. 4.**
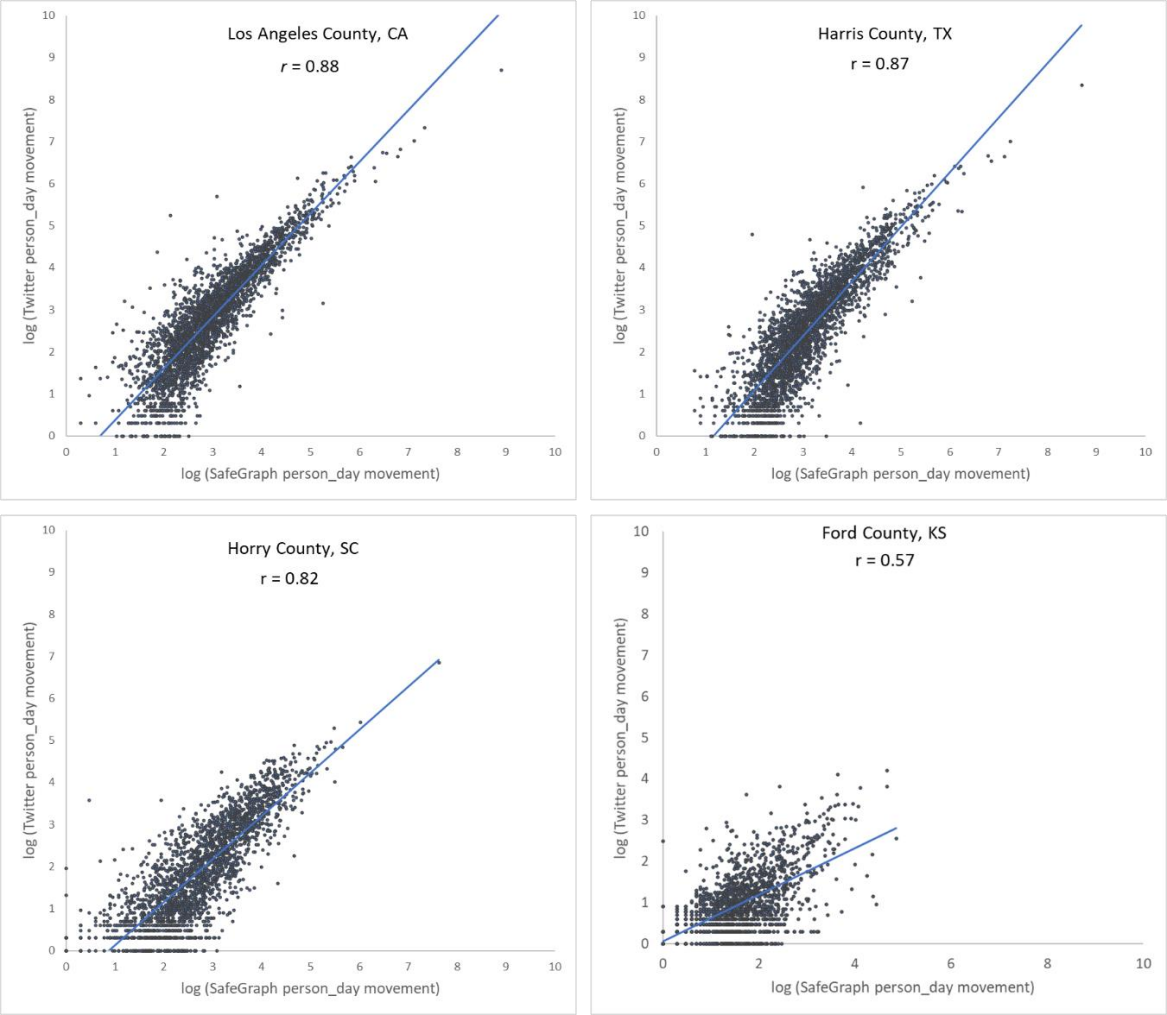
Scatter plots of log Twitter-derived person day movements and log SafeGraph-derived person day movements in 2019 for the four selected counties with varying populations. (a) Los Angeles County, California (CA), including Los Angeles metropolitan area. 2019 population: 10.04 million; (b) Harris County, Texas (TX), including Houston city. The most populous county in TX. 2019 population: 4.71 million; (c) Horry County, South Carolina (SC), including the popular beach destination Myrtle Beach. 2019 population: 354,081; and (d) Ford County, Kansas (KS), including the small Dodge City. 2019 population: 33,619. Population data were derived from the American Community Survey (ACS) 5-Year Data (2015–2019). US Census (2019).

To further examine the associations between the two movement datasets and the impact of county population size on the associations, we selected four counties with different geographical contexts and populations ranging from 3,300 to 10,000,000, and plotted the Twitter-derived person day movements and SafeGraph-derived person day movements in 2019 for each county. The scatter plots (Fig. 4) reveal a quasi-linear positive pattern for all four counties. Consistent with Fig. 3, the *r* value decreases as population decreases for the four counties of Los Angeles County, CA (0.88), Harris County, TX (0.87), Horry County, SC (0.82), and Ford County, KS (0.57). Notably, we observed only a slight drop in *r* (from 0.88 to 0.82) for Horry County with a relatively small population of 354,081. The findings indicate that geotagged Twitter-derived movement has a strong linear association with SafeGraph-derived population movement and reinforce that geotagged tweets can well capture population movements among places (counties in this study).

### Comparing PCI with Facebook SCI

We contrasted the PCI for each of the US counties with the Facebook Social Connectedness Index (SCI) data (Bailey et al., 2018a). This comparison allows us to evaluate the hypothesis that places connected through (social media) friendship links are likely to have more physical interactions (e.g., population movement). This hypothesis has already been suggested in recent studies (Kuchler et al., 2021), but not corroborated using SCI data. Thus, demonstrating this connection is relevant for many reasons, such as understanding spatial behavior under normal circumstances (e.g., business or commercial relationships, tourism, and migrations) or during extraordinary events such as a pandemic (e.g., the spread of infectious diseases) or a natural hazard (e.g., evacuation corridors).

As a measure of social connectedness based on friendship links on Facebook, SCI revealed that the majority of these links are found within 100 miles, showing an intense distance decay effect (Bailey et al., 2018b). The hypothesis of a positive association between social and spatial connections makes intuitive sense and helps understand population dynamics at different scales. To evaluate this, we first analyzed the correlation between PCI and SCI using all county pairs that had both PCI and SCI values (*n* = 1,702,531). Log transformation was used to address the highly skewed distribution of the PCI and SCI values among counties. Note that PCI values were multiplied by 1000 before taking the log to avoid negative values. The overall *r* of 0.62 indicates a strong linear association between social and spatial connections.

**Fig. 5.**
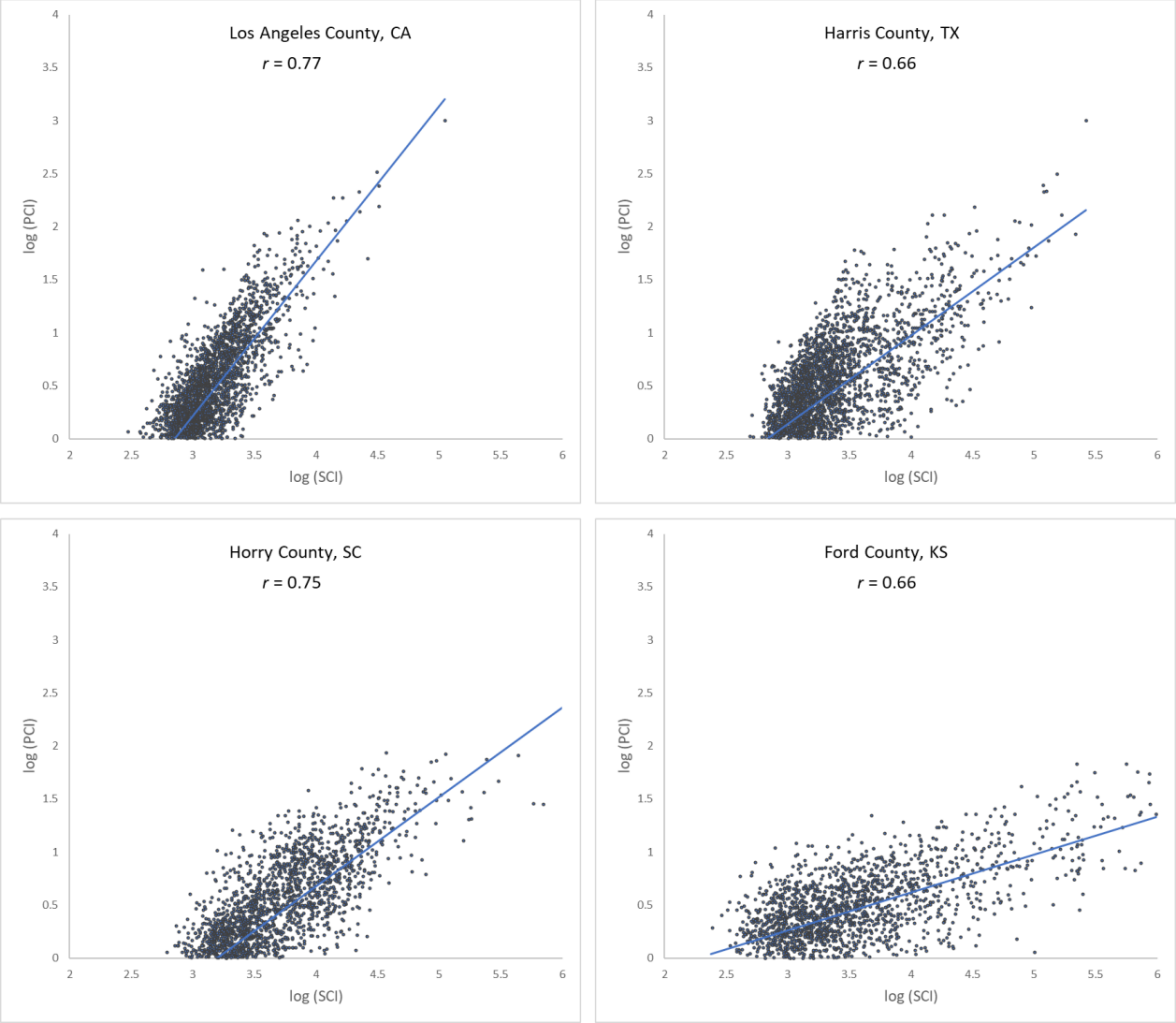
Scatter plots of log PCI and log PCI for the four counties

**Fig. 6.**
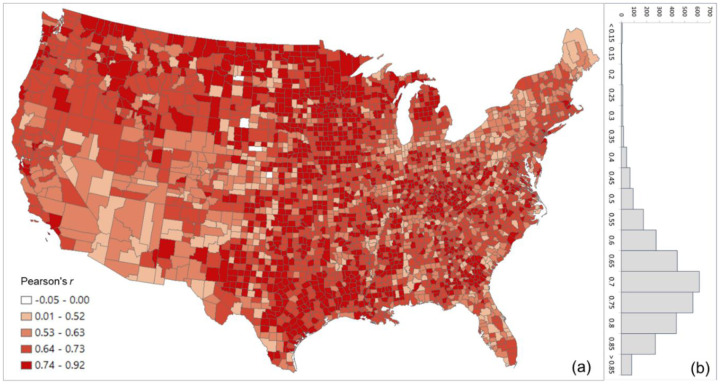
Distribution of the Pearson’s *r* between log PCI and log Facebook SCI for all counties(a) Spatial distribution; (b) histogram

Fig. 5 shows the scatter plots of log PCI and log SCI in 2019 for the four counties used in the previous section, further confirming the association of a measure of social connectedness with an index of spatial connectivity. The scatter plots also reveal that the association between SCI and PCI are not always stronger in more populated counties (e.g., *r* for Harris County is 0.66 while for the less populated Horry County it is 0.75). Another interesting observation is that the slope of the best-fit line is higher in more populated counties (e.g., Los Angeles County) than in lowly populated areas (Ford County), indicating that the same amount of change in friendships (SCI) is associated with larger change in people’s movement (PCI) in more populated counties, vice versa. To further examine the variations of such association among counties, we computed the Pearson’s *r* between PCI and SCI for each county to other counties. Fig. 6a shows that strong correlations are generally clustered in Midwest US, Texas, and Southeast Georgia. The histogram (Fig. 6b) shows the most repeated *r* ranging between 0.70 and 0.75.

The strong association between PCI and SCI confirms the hypothesis that regions connected through (social media) friendship links are likely to have more physical interactions. However, our findings also suggest caution about the relationship between these two variables. Although PCI and SCI are associated, one cannot substitute one for the other, as they represent different phenomena: social versus spatial behavior. More studies are needed to better understand the driving forces (e.g., urban-rural, demographic, and socioeconomic factors) behind the associations of the two variables. We believe PCI is an important addition, as it involves a new standardized measure of spatial connectivity based on population movement.

### Distance Decay Effect

**Fig. 7.**
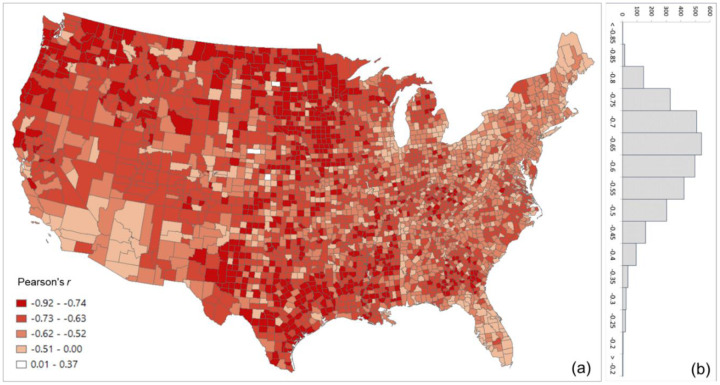
Distribution of the Pearson’s r between log PCI and log distance for all counties(a) Spatial distribution, (b) histogram

**Fig. 8.**
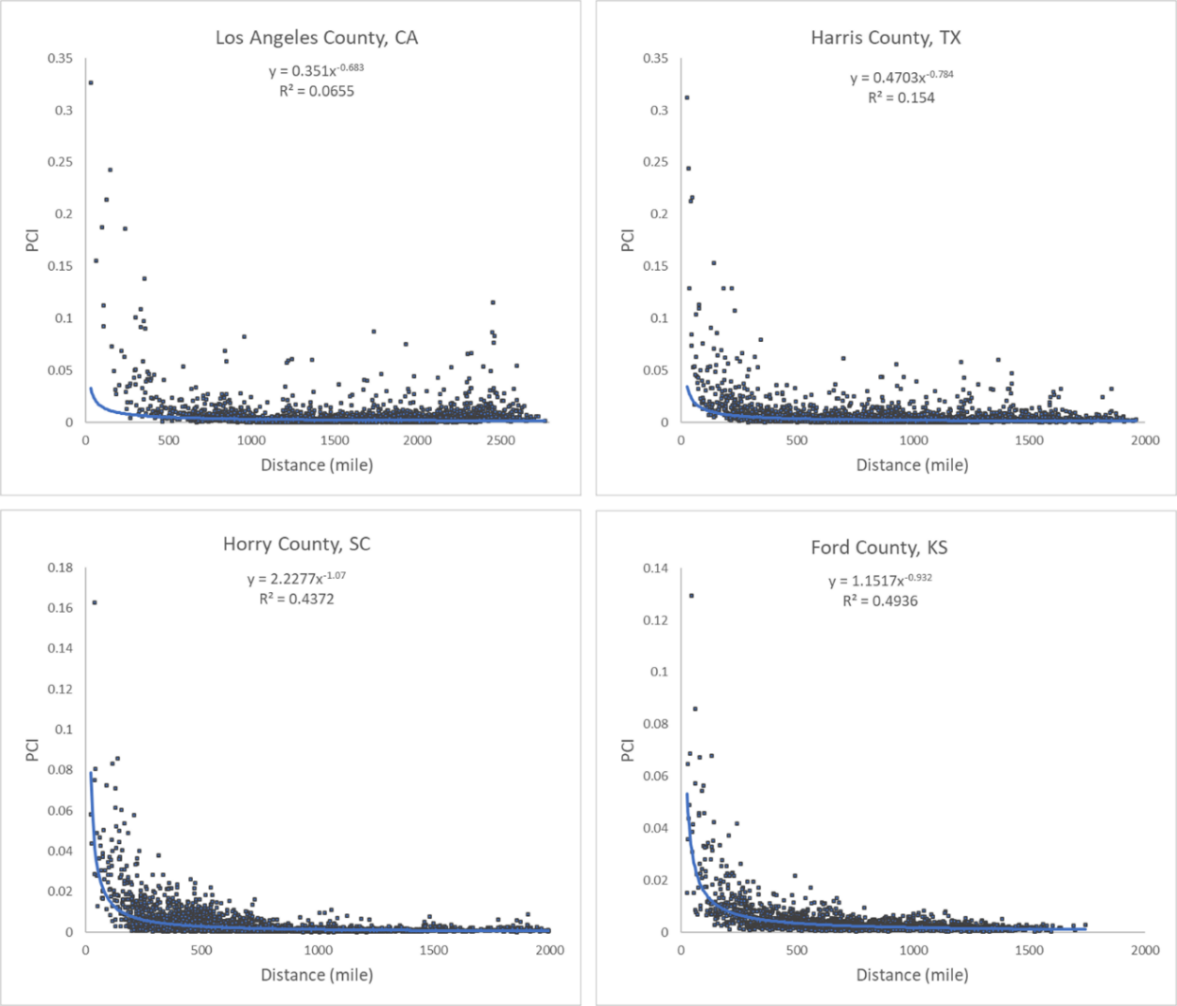
Scatter plots of PCI and distance for the four counties.

Our analysis revealed that PCI expresses a clear distance decay effect. In other words, the spatial connectivity between two distant places is likely to be lower than that observed between two near counties. However, there are some nuances in this broad assertion. Fig. 7 illustrates the association between log PCI and the log distance for each county to all other counties. The map (Fig. 7a) shows that less populated (rural) areas of the Midwest, Pacific northwest, or Texas have a stronger negative association between PCI and distance, meaning that these communities are more tightly knit with surrounding areas than with more distant communities (stronger distance decay effect). This phenomenon is also reflected in Fig. 8, where *R*^*2*^ values of the power law function decrease dramatically from lowly populated Ford County (0.494) to Harris County (0.154) to highly populated Los Angeles County (0.065). Pearson’s *r* was not used as the scatter plot, as the relationship is nonlinear. It should be noted that population size is likely a compounding factor that goes along with urban centers (e.g., metropolis) with large airports.

**Fig. 9.**
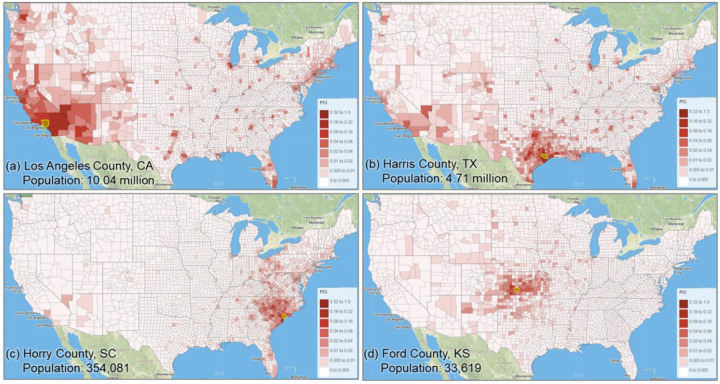
The selected four counties (highlighted with yellow boundaries in the maps) and their PCIs with other counties in the contiguous US. Population data were derived from ACS 5-Year Data (2015–2019) and US Census (2019).

The maps in Fig. 9 depict how the selected four counties are connected to other counties based on the PCI, which agrees with the above observations. On the other hand, Fig. 9 also shows that highly populated and touristy urban areas (well connected through airports), such as New York City, Miami, Orlando, Chicago, or Las Vegas, act as poles of attraction for people from distant locations. This is clear in Fig. 9a, where we can see how Los Angeles County, for instance, is more closely linked through spatial interactions with the New York City metropolitan area than with some California or Nevada counties. This behavior is also easily detected in Fig. 8 through the outliers of the point distributions in Los Angeles County.

### State Boundary Effect

Inspired by Bailey et al. (2018b), we also considered the effect of state borders shaping spatial connectivity. A higher PCI between a county pair indicates a strong relationship geographically. In a general sense, people tend to travel to their adjacent counties more frequently than non-adjacent counties. However, do the residents near the state border prefer the in-state counties as their destinations rather than the adjacent county across the state border? Or are the out-of-state counties more attractive? If state borders have a role in explaining spatial connectivity, people will tend to travel more within their home states than in neighboring states, even when the distance is fixed.

To evaluate the state boundary effect for each of the four counties, we first ran a linear regression with the following variables: the distance between the county and all other counties in contiguous US (*distance*), a categorical variable (*same_state*), and PCI (as the dependent variable). The result indicates that *same_state* variable shows a strong positive effect (*p* < .001) on PCI even after controlling for distance (Table 1). This implies that these four counties are more tightly (spatially) connected with other counties within the same state, even when compared to nearby counties in other states.

To test whether existing state borders are similar to the borders formed when we grouped together the US counties into communities (clusters) based on their spatial connectivity (i.e., PCI), we used a hierarchical agglomerative linkage clustering method following Bailey et al. (2018b) to create such homogenic spatial connectivity communities and compare them with the state administrative division of the US. Hierarchical agglomerative clustering groups county pairs based on their distance in feature space. In our experiment, the “distance” is defined as the inverse of PCI, which means a low PCI in a county pair has a long distance, and vice versa. In the beginning, every county is viewed as a separate community, and the two closest communities are combined into a new community. Distances of combined communities will be updated by the average of distances between county pairs of community pairs. The clustering stops when all counties are combined into a target number of communities. We chose 20, 50, and 75 clusters as the targeted number of communities.

**Fig. 10.**
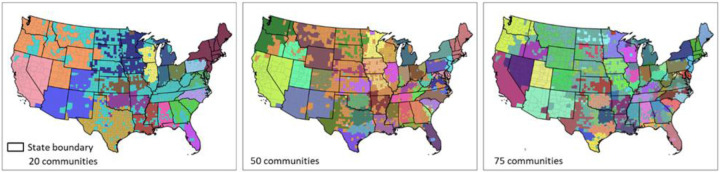
Results of the hierarchical agglomerative clustering of PCI with three different targeted numbers of communities for the contiguous US.

As shown in Fig. 10, most resulting distinct communities in the three maps are spatially contiguous, revealing the strong spatial connectivity of neighboring counties, an obvious consequence of spatial proximity. However, the resemblance of these three maps with state boundaries is quite remarkable across many areas, supporting the assertion that state boundaries do play a decisive role in shaping the spatial behavior of the population. For example, we can see how several clusters in the southwest US are essentially the state boundaries. Also, many other smaller clusters also respect the actual state boundaries. This pattern holds in the three maps with different cluster sizes.

A strong state boundary effect was also observed in social connectivity with Facebook SCI (Bailey et al. 2018b). These two findings are likely to be related; however, we do not know which one drives the other or if there are other variables conditioning this behavior (e.g., socio-spatial factors based on institutional or administrative circumstances). Further studies are needed to better understand the state boundary effect of PCI and its connections with SCI.

## Applications

PCI can potentially be applied in diverse fields that can benefit from a better understanding of human movement at varying spatial scales, such as infectious disease spread in public health, transportation, tourism, evacuation, and economics. Two examples are provided to exemplify how PCI can be used as a potential factor in analyzing and predicting infectious disease spreading and hurricane evacuation destination choice.

### PCI as a Factor in Predicting the Spatial Spread of COVID-19 During the Early Stage

Westchester County is an early (March 2020) hotspot of COVID-19 in the US (Hogan et al., 2020). Early confirmed cases and a high infection rate to family and friends increased social tension that residents from Westchester and surrounding areas were reportedly fleeing away (Tully and Stowe, 2020). In this application example, we explored the relationship between the spread of COVID-19 in the US and PCIs for Westchester County, NY.

**Fig. 11.**
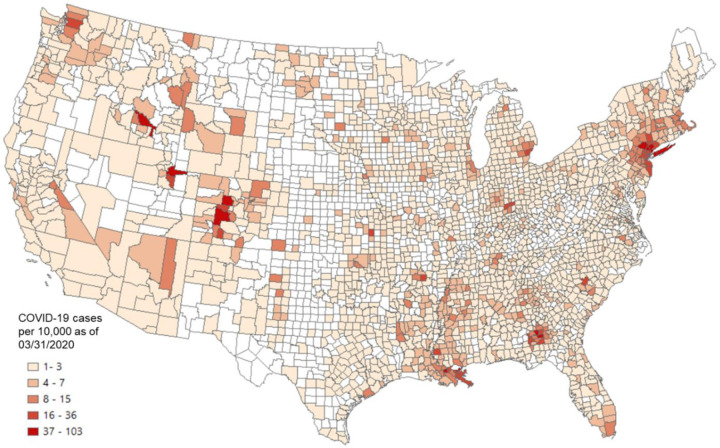
County level COVID-19 cases per 100,000 people as of March 31, 2020. COVID-19 case data were downloaded from NYT Github (New York Times, 2020). The county population was retrieved from the ACS five-year estimates (2014–2018).

Given that the incubation period of COVID-19 is about two to three weeks (Lauer et al., 2020), the number of cases confirmed before the end of March was used in the later calculation to capture the spread of COVID-19 in early and mid-March. Fig. 11 shows the county-level infection rate (number of confirmed cases per 10,000 people) as March 31, 2020. The number of confirmed cases is based on the New York Times (2020) database, and the total county population is based on the ACS five-year estimation (US Census, 2019). Dark red spots show the hotspots of COVID-19 confirmed cases. Westchester County and surrounding New York City areas were the main hotspots at the end of March.

To explore whether outbreaks of COVID-19 in the US are related to people who fled away from New York City in early March (Carey and Glanz, 2020), we used a linear regression model to examine the relationship between COVID-19 infection rate (as a dependent variable) and the connectivity between a given county and Westchester County using four measurements, including PCI computed with 2018 and 2019 Twitter data, respectively, Facebook SCI as of August 2020, and 2020 SafeGraph movement data (the person-day movements computed with the method in Appendix B using data from January to March, 2020). Table 2 shows the results of the four linear regression models. For all four measurements, positive relationships are significant at the 0.01 level. Among these four measurements, PCI for both 2018 and 2019 showed the highest adjusted *R*^*2*^ of 0.24 for both years. In other words, 24% of the variance of COVID-19 infection rate in each observed county can be explained by PCI alone. SafeGraph movement results in an adjusted *R*^*2*^ of 0.13. Facebook-based SCI shows the lowest adjusted *R*^*2*^ of 0.08, though the coefficient is still significant (*p <* 0.01).

Regression models controlling for the effect of geographic distance were also conducted with the four human mobility measurements. Results show that all four measurements still show significant positive correlations with the COVID-19 infection rate (*p* < 0.01; Table 3). The adjusted *R*^*2*^ for SafeGraph-derived movement and Facebook SCI remain unchanged, and the coefficient of the distance variable is not significant (*p* > 0.1). The adjusted *R*^*2*^ for both 2018 and 2019 PCIs only slightly increased by 0.01, from 0.24 to 0.25. While the distance variable is significant in these two models, its impact on the infectious rate is relatively weak given the small coefficient values (*β* = 0.00087 for 2019 PCI and *β* = 0.00091 for 2018 PCI).

This application demonstrates that PCI, computed from historical Twitter data, is a promising indicator in predicting the spatial spread of COVID-19 during the early stage, outperforming more current Facebook SCI (data as of August 2020) and SafeGraph-derived person-day movement data (from January 1 to March 31, 2020). We remark that PCI calculated with historical Twitter data of either 2018 or 2019 exhibits similar performance in the two models, suggesting the stability of place connectivity measured by PCI.

### PCI as a Factor in Predicting Hurricane Evacuation Destination Choices

Evacuation of coastal residents has been an effective and important protective action before the arrival of a hurricane (Cutter and Smith, 2009). Understanding where coastal residents are evacuating helps in evacuation route planning and resource allocations (Cheng et al., 2008). Residents of a county are likely to evacuate to a county where they have established relationships (friends, colleagues, familiar lodging stays, etc.). The preexisting relationships would be expressed by the PCI or SCI. In this section, we examined the association between PCI (computed using the 2019 Twitter data) and people’s evacuation destination choice using Hurricane Matthew in 2016 as a case study. We hypothesize that people are more likely to evacuate to a county that has a high PCI with the evacuation county. For comparison, we also tested the hypothesis that people are more likely to evacuate to a county that has a high SCI with the evacuation county.

**Fig. 12:**
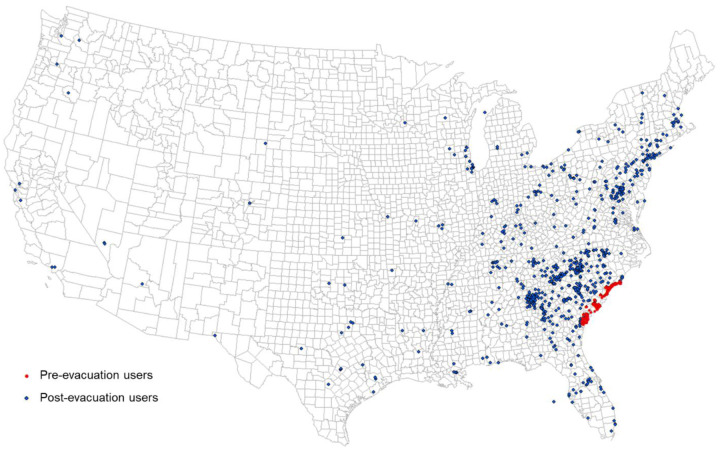
Hurricane Matthew Evacuation Estimation Using Geotagged Tweets. Red dots indicate user locations during the pre-evacuation period (October 2–4, 2016). Blue dots show user locations during the post-evacuation period (October 7–9, 2016).

Hurricane Matthew was a category 5 hurricane that visited the east coast of the US at category 1 in early October 2016. Evacuation orders for coastal counties under potential impact were placed by the governors of Georgia, South Carolina, and North Carolina on October 4, 2016. Twitter users were selected as individual evacuees for testing our hypothesis. The evacuation identification procedure followed the study area and evacuation timeline determined by Martin et al. (2017) and Jiang et al. (2019). In this study, we identified 272 evacuated individual Twitter users from Chatham County, GA, and 241 evacuated users from Charleston County, SC. All selected users had evacuated more than 50 miles away from their original coastal counties, and all of their destinations were not in the potential impact zone. The 272 evacuated individuals leaving Chatham County ended up in 120 destination counties, and the 241 Charleston County evacuees ended up in 118 destination counties (Fig. 12).

To test our hypotheses and the potential of PCI in predicting evacuation destination choice, we used a linear regression to model the relationship between the number of evacuated users in the destination counties (dependent variable) and PCI of the county pairs between Charleston County (origin) and each of the destination counties (*n* = 118). Distance between the evacuation county and each of the destination counties were used in the regression model as controls. SCI was tested by replacing PCI in the regression model for comparison. The same model configuration was used for Chatham County (*n* = 120). Table 4 shows the regression results for the four models.

For both counties, PCI shows a significant positive association with evacuee counts (*p* < 0.01). SCI shows a significant positive association with evacuee counts for Charleston County (*p* < 0.01), but the coefficient is not significant for Chatham County (*p* > 0.1). The distance variable is not significant for all four models (*p* > 0.1). The PCI model for Charleston County has an adjusted *R*^*2*^ of 0.71, indicating 71% variance can be explained by PCI. However, the adjusted *R*^*2*^ value for SCI has a much lower value of 0.29. For Chatham County, the PCI model has an adjusted *R*^*2*^ of 0.47, while the adjusted *R*^*2*^ value for the SCI model is only 0.04. This application demonstrates the potential of using PCI as a factor in modeling hurricane evacuation destination choice. The comparison of PCI and SCI shows that PCI outperforms SCI in this application scenario.

## Discussions

In this increasingly connected world, new places within cities or new types of relations across different places may emerge. Human societies comprise many interrelated contexts where individuals influence and interact with each other over space, time, and networks. Real-time social media data provide a promising opportunity that allows researchers and professionals to detect how places in cities are preferred, used, and related. Tweets capture a considerable portion of the activities, events, and issues about our society at a fine space-time scale. They reflect not only the socioeconomic activities that happen in our physical world, but also some cultures, human interests, and public concerns that exist only in the perceptions of people. The geotagged Twitter data allow us to detect locations where people visit. The sample size is much larger than that of conventional surveys. Such large samples can capture the behavior of population groups that are not easily accessible and hence difficult to be involved in conventional surveys, particularly those underrepresented neighborhoods. Tweets allow a more natural and direct sharing of information in contrast to surveys in which the subjects may feel that their behavior is monitored.

This research employs massive, geotagged Twitter data to delineate the spatial interactions between places by developing PCI. The results show that tweets can be used to reveal place connectivity, which also has strong correlations with other data streams such as SafeGraph and Facebook. Compared to the latter data sources, Twitter data are more openly available over time and at the individual level, which depicts the robust connection between locations from the human dynamics level. Given the increasing dynamic interplay among places and in particular cities, PCI provides invaluable opportunities to explore human behavior and social phenomena from the individual perspective to the population level. As demonstrated by the two application examples, PCI can be used for research in infectious disease and hurricane evacuation that benefit from a better understanding of human movement.

Cities grow and decline in an interdependent system of flows, connections, and relations. As Beaverstock et al. (2000) stated, “the world-cities literature is seriously unbalanced: it has a surfeit of interesting theoretical concepts for treating the nodes of the world-city network, but these exist alongside a deficit in empirical concern for measuring relations between the nodes” (p. 126). PCI provides one approach for representing the idea that the world should be viewed as networks rather than the mosaic of cities (Beaverstock et al., 2000), and offers promising opportunities to measure and compare the flows between cities. Also, scholars of regional science, urban studies, and geography have long been interested in the spatial interactions between cities and regions as they convey the spatial structure of a region. PCI can be jointly linked to emerging geotagged data, such as Yelp and Transportation Network Company data, to reveal a more complete picture of spatial structure dynamics. Combined with PCI, the place hierarchy and spatial clusters can be revealed based on both virtual and physical interactions.

Although the outcome of the behavior of PCI largely matches our expectations and with the results of other big social data sources, using social media data to identify spatial interaction has the following limitations. First, research using social media data has been criticized for being biased for representing all populations. For example, young adults are more likely than their older counterparts to use Twitter (Malik et al., 2015; Jiang et al., 2018). Second, geotagged tweets are unevenly distributed across space and time, causing data sparsity and measurement reliability issues. Third, the correlation between PCI and other indicators from social networking platforms is context-sensitive. Hence, the results may not be readily generalized or used for prediction in other places. Fourth, episodic events, such as holidays and hurricanes, would largely attract/hinder users’ movement to specific places, which might distort the connectivity if data is only collected for short periods. These episodic events will affect the accuracy and consistency of measurement results. This issue can be addressed by computing PCI over a relatively long period (e.g., one year). Lastly, Internet access and governmental policies on social media will also affect the reliability of such measurements.

## Conclusions

Documenting and understanding human spatial interaction has been an active domain in not just geography but other space-related social sciences for decades. Place relationships are believed to be shaped by dynamic human movement, whose intensity further quantifies the connectivity (strength of the linkages) among places. With the advances in technologies in the past decades, the connectivity among places is ever-evolving dynamically, thus demanding spatiotemporal-continuous observations with harmonized approaches. Fortunately, the emergence of big social media data, benefiting from the advent of geopositioning techniques and the popularity of social media platforms, offers a new venue where collecting human spatial interactions becomes less-privacy concerning, easily assessable, and harmonized.

In this study, we proposed a place connectivity index based on people’s spatial interactions among places revealed from geotagged Twitter posts. Defined as the normalized number of Twitter users who shared spatial interactions during a specified time period, the proposed PCI is a harmonized and easy-to-understand place connectivity metric, expected to benefit various domains requiring knowledge in human spatial interactions. The interactive web portal aims to facilitate place connectivity visualization and provide downloadable connectivity matrices to support research needs.

To better understand the characteristics of PCI, we conducted a series of experiments using PCI and other data sources. An overall Pearson’s *r* of 0.71 between the population movement derived from Twitter and SafeGraph (10% penetration in the US population) reveals that geotagged tweets can well capture the population movement at the US county level. The comparison between PCI and Facebook SCI (a popular connectivity index based on social network) with an overall *r* = 0.62 suggests a strong connection between spatial interactions and social interactions, confirming the hypothesis that “regions connected through many friendship links are likely to have more physical interactions between their residents” (Kuchler, Russel & Stroebel, 2021). Like many connectivity measurements that are bounded by the first law of geography, we found that PCI generally follows distance decay form, while the distance decay effect is found weaker in more urbanized counties with a denser population. This phenomenon can be explained by the existence of long-distance transportation facilitates (e.g., airports, railways, and bus stations) that, to some extent, express a hierarchical diffusion relationship rather than a contagious diffusion. We further observed a strong state boundary effect in PCI, indicating that counties in the same state are more connected, evidenced by their higher PCI values.

We demonstrated that PCI could address real- problems requiring place connectivity knowledge using two applications: 1) modeling the spatial spread of COVID-19 during the early stage and 2) modeling hurricane evacuation destination choices. In the first application, we found that the proposed PCI for Westchester County, NY, an early hotspot of COVID-19 in the U.S., could explain 22% of the variance in COVID-19 cases among U.S. counties at the early outbreak, which was much higher than Facebook SCI (8%) and the population movement derived from SafeGraph (13%). In the second application, we found that PCI explains a considerably higher percentage of variance in local residents’ choices of destination county during 2016 Hurricane Matthew compared with Facebook’s SCI, suggesting the superiority of spatial interactions in modeling evacuation choices than social interactions.

With the effects of geographic distance being weakened by technological advances, place connectivity quantified by human spatial interactions has been evolving since the very first day of modern society and will continue to evolve at an accelerating pace in the future. Taking advantage of the growing popularity of social media, the PCI proposed in this study contributes to a scale-free, spatiotemporal-continuous measurement of place connectivity, benefiting numerous applications such as infectious disease modeling, transportation planning, evacuation modeling, tourism management, to list a few. The methodological and contextual knowledge of PCI, together with the launched visualization platform and data sharing capability, is expected to support research fields in need of prior knowledge in human spatial interactions.

## Figures and Tables

**Table 1:** Regression Results for the Four Counties

	Los Angeles County		Harris County		Horry County		Ford County	
	Coefficient	SE	Coefficient	SE	Coefficient	SE	Coefficient	S.E.
Intercept	0.0059***	0.0008	−0.0075***	0.0007	0.0078***	0.0003	−0.0073***	0.0003
Same state	0.0495***	0.0017	0.0170***	0.0010	0.0273***	0.0010	0.0170***	0.0006
Distance	−8.3E-07	−4.5E-07	−3.6E-06***	6.9E-07	−4.5E-06 ***	2.5E-07	−4.9E-06 ***	3.7E-07
Adjusted *R*^*2*^	0.24		0.16		0.33		0.45	
Observations	3008		2932		2446		1788	

Note:

* p < 0.1

** p < 0.05

*** p < 0.01.

The county centroid was used for the distance calculation between two counties (distance unit: mile).

**Table 2:** Regression Result Using COVID-19 Infection Rate as the Dependent Variable, and PCI, SCI, or SafeGraph as the Predictor Variable

	2019		2018		Facebook (2020)		SafeGraph (2020)	
	PCI		PCI		SCI		Person-day movement	
	Coefficients	SE	Coefficients	SE	Coefficients	SE	Coefficients	SE
Intercept	1.63496***	0.11603	1.60351***	0.12215	2.26827***	0.12262	2.47287***	0.13615
PCI/SCI/SafeGraph	0.22505***	0.00931	0.21112***	0.00901	0.00013***	0.00001	0.00040***	0.00003
Adjusted *R*^*2*^	0.24		0.24		0.08		0.13	
Observations	1847		1755		1847		1497	

* p < 0.1

** p < 0.05

*** p < 0.01

**Table 3:** Regression Result Using COVID-19 Infection Rate as the Dependent Variable, and PCI, SCI, or SafeGraph as the Predictor Variable Controlling for Distance

	2019		2018		Facebook (2020)		SafeGraph (2020)	
	PCI		PCI		SCI		Population movement	
	Coefficients	SE	Coefficients	SE	Coefficients	SE	Coefficients	SE
Intercept	0.73883***	0.22272	0.67638***	0.23186	2.19947***	0.23329	2.43267***	0.24346
PCI/SCI/SafeGraph	0.23706***	0.00960	0.22307***	0.00931	0.00013***	0.00001	0.00040***	0.00003
Distance	0.00087***	0.00019	0.00091***	0.00019	0.00007	0.00020	0.00004	0.00022
Adjusted *R*^*2*^	0.25		0.25		0.08		0.13	
Observations	1847		1755		1847		1497	

* p < 0.1

** p < 0.05

*** p < 0.01

**Table 4:** Regression Results for the Number of Evacuated Users in the Destination Counties (Dependent Variable) and PCI (or SCI) of the County Pairs Between Evacuation County and Each of the Destination Counties

	Charleston County		Charleston County		Chatham County		Chatham County	
	PCI		SCI		PCI		SCI	
	Coefficients	SE	Coefficients	SE	Coefficients	SE	Coefficients	SE
Intercept	0.28676	0.23274	1.63200***	0.32877	−1.66645**	0.63781	2.57697***	0.69610
PCI/SCI	0.09611***	0.00606	0.00003***	0.00000	0.19071***	0.01927	0.00001	0.00001
Distance	0.00019	0.00030	−0.00047	0.00047	0.00115	0.00083	−0.00155	0.00112
Adjusted *R*^*2*^	0.71		0.29		0.47		0.04	
Observations	118		118		120		120	

* p < 0.1

** p < 0.05

*** p < 0.01

## Appendix A

### Computation of US County Level PCI Using 2019 Geotagged Twitter Data

Worldwide geotagged tweets were collected in 2019 using the public Twitter Stream Application Programming Interface (API). A total of 391,503,203 geotagged tweets from 4,892,458 distinct Twitter users were extracted within the bounding box covering the contiguous United States. These tweets are geotagged at different geographic levels. About 80% of tweets are tagged at the place level, such as a city, neighborhood, and point of interest (POI), and the rest are geotagged with latitude and longitude. For computing PCI at the county level, we filtered out tweets geotagged at a geographic level lower than a city (e.g., state level). If a tweet is geotagged at the place level, the coordinates (latitude and longitude) of the place centroid was used in the analysis. Following Martin et al. (2020), we filtered out the non-human tweets (e.g., automated weather reports, job offers, and advertising) by checking the tweet source from which application a tweet is posted. For example, tweets automatically posted for job offers from the source TweetMyJOBS and CareerArc are removed. After spatial filtering and non-human filtering, a total of 316,797,441 tweets remained from 4,609,040 unique Twitter users. The process was performed using Apache Impala in a Hadoop environment.

After data cleaning, each Twitter user was assigned to a county based on that user’s tweet location daily. For example, if a user tweeted in three counties on a specific day, then for that user on that day, three rows were generated. After processing all users, counties, and days in 2019, a big table (*CountyUserDate*) with three fields (*county, user, date) was generated,* including 95,701,425 county-user-date tuples. The daily table was then aggregated along the date to produce a new table (*CountyUserDays*) with three fields (*county, user, days*), where *days* indicates the number of days (in 2019) a user was observed in a county, including 11,875,433 county-user-days tuples. Based on the *CountyUserDays* table, for each county, two numbers were derived: 1) the number of shared users with other counties and 2) the number of total observed users in each county. Finally, PCI was computed for each county pair (those with shared users) using Eq. 1. This process was conducted using Apache Hive coupled with Esri GIS tools for Hadoop (Esri, 2019).

## Appendix B

### Computation of the County Person-Day Movements Using 2019 SafeGraph Data

The SDM data were downloaded from SafeGraph and loaded to our Hadoop cluster. There were 23 fields in the SDM table, and three were used to derive the population movement, including *origin_census_block_group*, *destination_cbgs*, and *date_range_start*. The *origin_census_block_group* is the unique 12-digit FIPS code for the Census Block Group. *Destination cbgs* contains a list of key-value pairs with key indicating the destination census block group (from the origin census block group) and “value is the number of devices with a home in *census_block_group* that stopped in the given destination census block group for >1 minute during the time period” (https://docs.safegraph.com/docs/social-distancing-metrics). The *date_range_start* was used to extract the date information.

Based on the three fields, we generated a new table (*SgDailyOD*) with each row showing the number of devices from an original block group to a destination block group on a specific day, resulting in over 6 billion (6,144,802,397) origin-destination flows. Based on the *SgDailyOD* table, we further aggregated the flows to the county level for 2019, generating a new table (*SgCountyPersonDayMovement*) with each row showing the total number of device movements between two counties on all days of 2019, resulting in over 6 million county pairs (6,119,765). Note that the number of movements includes the movements from both directions. For instance, if there are *m* number of movements from county A to county B, and *n* number of movements from county B to county A, then the number of movements between the two counties is calculated as *m + n*. The entire process was conducted in our in-hour Hadoop cluster using Apache Hive and Impala.

## Appendix C

### Computation of the County Person-Day Movements Using 2019 Geotagged Twitter Data

After data cleaning, each Twitter user was assigned to a county based on that user’s tweet location daily. For example, if a user tweeted in three counties on a specific day, then for that user on that day, three rows were generated. After processing all users, counties, and days in 2019, a big table (*CountyUserDate*) with three fields (county, user, date) was generated, including 95,701,425 county-user-date tuples. This step was conducted using Apache Hive coupled with ESRI tools for Hadoop. The person-day movement with geotagged tweets was calculated by aggregating the *CountyUserDate* table along the date, generating a new table (*SgCountyPersonDayMovement)* with each row showing the total number of user movements between two counties on all days of 2019, resulting in over 3 million (3,405,113) county pairs.
